# Nuclear expression of Snail1 in borderline and malignant epithelial ovarian tumours is associated with tumour progression

**DOI:** 10.1186/1471-2407-9-289

**Published:** 2009-08-20

**Authors:** Hanna Tuhkanen, Ylermi Soini, Veli-Matti Kosma, Maarit Anttila, Reijo Sironen, Kirsi Hämäläinen, Laura Kukkonen, Ismo Virtanen, Arto Mannermaa

**Affiliations:** 1 Pathology and Forensic Medicine, Institute of Clinical Medicine, University of Kuopio, PO Box 1627, FI-70211 Kuopio, Finland; 2 Department of Clinical Pathology, Kuopio University Hospital, PO Box 1777, FI-70211 Kuopio, Finland; 3 Biocenter Kuopio, University of Kuopio, PO Box 1627, FI-70211 Kuopio, Finland; 4 Department of Obstetrics and Gynecology, Kuopio University Hospital, PO Box 1777, FI-70211 Kuopio, Finland; 5 Institute of Biomedicine/Anatomy, University of Helsinki, PO Box 63, FI-00014 Helsinki, Finland

## Abstract

**Background:**

Transcription factor Snail1 has a central role in induction of epithelial-mesenchymal transition (EMT). The aim of the present study was to elucidate the expression of Snail1 protein during epithelial ovarian tumourigenesis and to study the association of Snail1 expression with clinicopathological factors and prognosis.

**Methods:**

Epithelial and stromal fibroblast-like fusiform cells of 14 normal ovarian samples, 21 benign, 24 borderline and 74 malignant epithelial ovarian tumours were studied for Snail1 protein using immunohistochemistry.

**Results:**

Nuclei of surface peritoneal cells of normal ovaries (*n *= 14) were regarded as negative for Snail1. Nuclear expression of Snail1 protein in epithelial ovarian tumours was increased during tumour progression from precursor lesions into carcinomas both in epithelial (p = 0.006) and stromal cells (p = 0.007). Nuclei of benign tumours (*n *= 21) were negative for Snail1. In borderline tumours (*n *= 24) occasional positive epithelial cells were found in 2 (8%) samples and in 3 (13%) samples stromal cells were focally positive for Snail1. In carcinomas (*n *= 74) focal Snail1 staining in epithelial cells was present in 17 (23%) tumours, and in stromal cells in 18 (24%) tumours. Nuclear expression of Snail1 in epithelial or stromal cells was not associated with clinicopathological factors or prognosis.

**Conclusion:**

Nuclear Snail1 expression seems to be related to tumour progression, and expression in borderline tumours indicates a role for Snail1 in early epithelial ovarian tumour development. Snail1 also appears to function more generally in tissue remodelling as positive staining was demonstrated in stromal cells.

## Background

Ovarian cancer is among the most common cancers in women worldwide and a common cause of cancer deaths [1]. It consists of several histological types: serous, mucinous, clear cell and endometrioid adenocarcinomas which all belong to epithelial tumours. Although the precursor lesion of epithelial ovarian cancer is still yet unidentified, the two-pathway model of ovarian cancer pathogenesis is gaining acceptance [2-4]. Type I tumours, i.e. low-grade serous ovarian carcinoma and mucinous ovarian cancers probably arise from benign adenomas and borderline tumours, and clear cell and endometrioid tumours may develop from endometriosis. Type II tumours, i.e. high grade serous ovarian cancer with aggressive phenotype is thought to arise directly from the surface epithelium of the ovary or from inclusion cysts.

Ovarian surface epithelial cells can convert to mesenchymal, fibroblast-like phenotype at least *in vitro *[5]. This epithelial-mesenchymal transition (EMT) is a complex cellular process during which cellular phenotype and function is changed [6]. EMT occurs in normal physiological processes during embryogenesis and wound healing. It has also been recognised in different pathological processes, e.g. in fibrosis and carcinomas [6]. Our previous results indicate that EMT occurs in epithelial ovarian carcinoma [7]. In addition to tumour invasion and metastasis EMT has been connected to early steps of carcinogenesis in epithelial malignancy [8,9].

Transcription factor Snail1 has been considered to have a key role in the induction of EMT [10]. Snail1 is a repressor of E-cadherin, an adherens junction protein maintaining cell-cell adhesion in epithelium. Snail1 may also enhance levels of other EMT inducing transcription factors such as ZEB-1 and ZEB-2 [11]. Due to expression of Snail1 and other EMT regulatory transcription factors epithelial cells lose many of their epithelial characteristics and take on the properties which are typical of mesenchymal cells. Snail1 may produce changes in cell shape, migration and invasion. It can regulate cell cycle progression and prevent cell proliferation by attenuating cyclin D2 transcription and increasing the expression of p21 [12]. Snail1 may also prevent apoptosis by activating MAPK and PI3K survival pathways [12].

Expression of Snail1 in epithelial cells of different malignant tumours has been investigated in several studies with dissimilar results [13]. Recently developed two monoclonal anti- Snail1 antibodies are well-characterised and show clear nuclear staining of the protein in different tumour types [11,14,15]. Using one of these antibodies, Blechschmidt et al. [16] have detected nuclear staining of Snail1 in 38% (18/48) of ovarian carcinomas and in 29% (25/87) of endometrial cancers [17]. Snail1 may be important in the pathogenesis of spindle cell carcinomas of the head and neck as nuclear staining has been reported in 63% (19/30) of cases [18]. However, lower nuclear staining has been detected in adenocarcinomas of upper gastrointestinal tract and other types of head and neck squamous cell carcinomas [15,18,19]. In addition, Snail1 staining in cell cytoplasm and/or nucleus has been observed in 16% (40/251) of laryngeal squamous cell carcinomas [20]. In ovarian cancer, Snail1 mRNA has been detected more frequently than protein since 93% (38/41) of primary tumours expressed mRNA [21]. Snail1 positive stromal cells have previously been detected in many adeno and squamous cell carcinomas [14-19,21]. However, there are no published data about Snail1 expression in precursor ovarian lesions.

To elucidate more properly the role of Snail1 in human epithelial ovarian tumour development we have analysed Snail1 protein in epithelial and stromal compartments of normal ovaries and in benign, borderline and malignant ovarian tumours by immunohistochemistry. In addition, to evaluate the prognostic value of Snail1 protein expression we have compared the results with known clinical data of the patients.

## Methods

### Patients

Our ovarian material consisted of 14 normal ovarian samples, 21 benign and 24 borderline epithelial ovarian tumours and 74 epithelial ovarian carcinomas (Table 1). The samples were collected in Kuopio University Hospital, Finland, between 1999 and 2005. Staging of primary tumours was based on standards of the International Union Against Cancer (UICC) [22]. Histological type and grade were evaluated according to the World Health Organisation (WHO) [23]. Patients who died because of any postoperative complications (deaths within one month after the operation) were not included in the survival analysis. Median follow-up time for all patients was 38 months (range 2–76 months). The research was approved by the ethical committee of Kuopio University and Kuopio University Hospital.

**Table 1:** Clinicopathological characteristics of the patients

Variable	Normal	Benign	Borderline	Carcinoma
Total	14 (100)	21 (100)	24 (100)	74 (100)
Median age at diagnosis, years^a^				59 [41–88]
Histologic subtype				
Serous		10 (48)^b^	12 (50)^b^	41 (56)
Mucinous		10 (48)^b^	12 (50)^b^	7 (9)
Endometrioid				17 (23)
Clear cell				7 (9)
Other		1 (4)^c^		2 (3)^d^
Histological grade				
1				9 (12)
2				32 (43)
3				33 (45)
Stage				
I				12 (16)
II				8 (11)
III				36 (49)
IV				18 (24)
Primary residual tumour				
None				30 (40)
≤ 1 cm				11 (15)
> 1 cm				33 (45)
Chemotherapy response				
No data				6 (8)
Complete response				52 (70)
Partial response				2 (3)
Stable disease				1 (1)
Progressive disease				7 (10)
No chemotherapy				6 (8)
Tumour recurrence				
No recurrence				20 (27)
Recurrence				31 (42)
No data				23 (31)
Patient status				
Dead, ovarian cancer				30 (41)
Alive				40 (54)
Unknown				4 (5)
Median follow-up time, months				38 [2–76]

Values are *n *(%) unless stated otherwise.

^a^Values in square brackets indicate range.

^b^Cystadenomas

^c^Brenner tumour

^d^Include two adenocarcinomas not otherwise specified.

### Immunohistochemistry

Five-μm-thick paraffin-embedded tissue sections of all ovarian samples were stained immunohistochemically. Shortly, after deparaffinisation and rehydration, the sections were heated in a microwave oven for 2 × 5 min in Tris-EDTA buffer (pH 9.0), incubated in a Tris-EDTA buffer for 20 min and washed twice for 5 min in phosphate buffered saline (PBS). Hydrogen peroxide (5%, 5 min) was used to block endogenous peroxidase, followed by washing with water 2 × 5 min and with PBS for 2 × 5 min. Non-specific binding was blocked with 1,5% normal serum in PBS for 35 min at room temperature. The sections were incubated over night at 4°C with the mouse monoclonal anti – Snail1 antibody (1:1000 dilution) [11,14]. Negative control was incubated with 1% bovine serum albumin in PBS. Next, the slides were washed with PBS for 2 × 5 min and incubated with the biotinylated secondary antibody (ABC Vectastain Elite Kit, Vector Laboratories, Burlingame, CA, USA) for 45 min at room temperature. After this, the slides were washed with PBS for 2 × 5 min, incubated for 50 min in preformed avidin-biotinylated peroxidase complex (ABC Vectastain Elite Kit, Vector Laboratories, Burlingame, CA, USA) and washed with PBS for 2 × 5 min. The colour was developed with diaminobenzidine tetrahydrocloride (DAP) (Sigma, St. Louis, MO, USA). The slides were counterstained with Mayer's haematoxylin, washed, dehydrated, cleared and mounted with Depex (BDH, Poole, UK).

### Evaluation of the stainings

Snail1 expression in all samples was analysed by two main observers (HT, LK) unaware of the clinical data and three senior pathologists (YS, V-MK, RS) evaluated a subset of the samples. The slides were first overviewed to find five hot spot regions (areas with the maximum number of positive cells per microscopic field) in the tissue studied. Stained epithelial/stromal cell nuclei in these hot spot regions were counted using 40 × objective [24]. For positive nuclear staining in stromal compartment of the tumours only fibroblast-like fusiform cells were included. The division of samples to negative or positive as regards to nuclear staining was assessed according to mean value. Among epithelial tumour cells the mean was 3 nuclei (range 0 – 49 nuclei) and among carcinoma associated stromal cells the mean was 4 nuclei (0 – 79 nuclei). Cytoplasmic staining of Snail1 in epithelium was graded as negative, when < 5% and positive, when ≥ 5% of the normal/tumour cells were positive for Snail1 [17].

### Statistical analysis

The statistical analyses were performed using SPSS 14.0 software (SPSS Inc., Chicago, IL, USA) except for testing trend. Mann-Whitney test was used to examine the relationship between continuous variables. A chi-squared test was used in analysing frequency tables. Interobserver agreement for the positivity of the stainings was evaluated by Spearman statistics. Univariate survival analyses were evaluated using the Kaplan-Meier method. The statistical differences between the curves were analysed using the log-rank test. Multivariate survival analysis was calculated using Cox's proportional hazards model. Disease related survival was defined as the time between the date of surgery and the date of death due to ovarian cancer. Recurrence free survival was defined by the time interval between the date of surgery and the date of recurrence. Trends were examined with chi-squared test for trend in proportions using R version 2.7.2 (The R Foundation for Statistical Computing).

## Results

**Figure 1:**
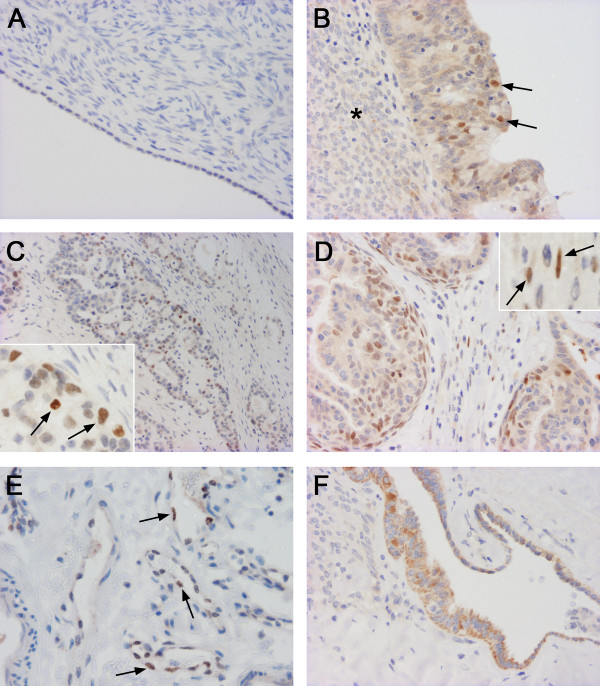
**Staining of Snail1 protein in ovarian samples**. a) A normal ovary with negative surface peritoneal cell nuclei (× 200). b) A serous borderline tumour with nuclear staining in some epithelial cells (arrow). Tumour stroma is indicated by asterisk. (× 200). c) A clear cell carcinoma with positive epithelial cell nuclei (arrows) (× 100). Insert (× 400). d) An endometrioid carcinoma with positive nuclear staining both in epithelial and stromal fibroblast-like fusiform cells (arrows) (× 200). Insert (× 400). e) A benign serous cystadenoma with nuclear staining in a subset of endothelial cells (arrows) (× 200). f) A benign serous cystadenoma with focal cytoplasmic staining in epithelial cells (× 200).

In *normal *ovaries (*n *= 14) surface peritoneal cell nuclei were regarded as negative for Snail1 (Figure 1a). There were no signs of the protein in stroma either.

In tumours an increased nuclear Snail1 content was associated with increasing tumour malignancy both in epithelial (p = 0.006) and stromal compartments (p = 0.007) (Table 2). In *benign *tumours (*n *= 21) all epithelial and stromal cell nuclei were negative. In *borderline *tumours (*n *= 24) nuclear positive epithelial cells were observed focally in 2 (8%) samples (Figure 1b) and stromal cells were focally positive in 3 (13%) samples.

**Table 2:** Staining of Snail1 protein in ovarian tumours

Tissue	Nucleus^b^	Cytoplasm^c^	Total
Benign			
Epithelium	0 (0)	19 (90)	21 (100)
Stroma^a^	0 (0)	-	21 (100)
Borderline			
Epithelium	2 (8)	20 (83)	24 (100)
Stroma^a^	3 (13)	-	24 (100)
Carcinoma			
Epithelium	17 (23)	32 (43)	74 (100)
Stroma^a^	18 (24)	-	74 (100)

Values are *n *(%).

^a^Fibroblast-like fusiform cells

^b^Epithelial nuclei p = 0.006 and stromal nuclei p = 0.007, chi-squared test for trend in proportions.

^c^Epithelial cell cytoplasm p < 0.001, chi-squared test for trend in proportions.

In *carcinomas *(*n *= 74) focal nuclear Snail1 immunoreactivity in epithelial cells was present in 17 (23%) samples (Figure 1c). In carcinoma-associated stromal cells the staining was focal and observed in 18 (24%) cases (Figure 1d). A good agreement was found between the evaluations in staining positivity between the observers (Spearman's correlation coefficient 0.599). No association was found between nuclear Snail1 expression in epithelium or stroma or between Snail1 expression and clinicopathological factors including histological subtype, grade, stage, primary residual tumour, chemotherapy response and tumour recurrence. Further, nuclear Snail1 expression was not related to overall survival or recurrence free survival. Nuclear staining of Snail1 protein in the subset of endothelial cells was demonstrated in normal ovaries as well as in benign, borderline and malignant tumours (Figure 1e).

In addition to nuclear staining also cytoplasmic Snail1 immunoreactivity was evaluated in epithelial cells of normal ovary and different tumour types. In epithelial inclusions of normal ovaries positive cytoplasmic staining was observed in 57% (8/14) of the samples. Decreased cytoplasmic expression of Snail1 in epithelial cells of the tumours was related to increasing tumour malignancy (p < 0.001). While 90% (19/21) of the benign tumours showed positive cytoplasmic staining 83% (20/24) of the borderline tumours and only 43% (32/74) of the carcinomas were positive (Figure 1f). Cytoplasmic expression in carcinoma cells did not associate with any of the analysed clinicopathological factors or nuclear staining in malignant cells.

## Discussion

Several lines of evidence show that EMT has a central role in invasion and metastasis of different types of tumours facilitating conversion of polarized, epithelial phenotype to motile fibroblastoid or mesenchymal phenotype [6]. Recently EMT markers have also been associated with early steps of tumourigenesis [8,9]. The aim of this study was to elucidate the role of transcription factor Snail1, an inducer of EMT, in the development of epithelial ovarian tumours.

According to our results nuclear expression of Snail1 protein seems to be a feature of tumour progression in epithelial ovarian tumourigenesis. Increased Snail1 expression was associated with increasing tumour malignancy both in epithelial compartment (p = 0.006) and in stromal compartment (p = 0.007). Our result is in concordance with previous studies indicating Snail1 as a regulator of EMT as well as literature describing it as an inducer of invasive phenotype [10]. However, Snail1 protein may have additional roles in different types of tumours.

Nuclei of all benign tumours were negative for Snail1. In line with our results one report presented negative Snail1 expression in mouse intestinal adenomas, and another in human hyperplastic oral mucosa [19,25]. However, dysplastic oral mucosa has been reported to show single distributed Snail1 positive cells [19]. We found similarly focally positive nuclear Snail1 protein staining in borderline ovarian tumours. Snail1 expression in precursor lesions may suggest a role for the protein in early ovarian tumourigenesis before invasion or metastasis. Its expression in borderline tumours might be related to other known functions of Snail1. Indeed, members of Snail1 family can also act as more general regulators of cell adhesion and movement in EMT-related or EMT-independent manner [26]. In cancer Snail1 has been described to act primarily as a survival factor and an inducer of cell movement rather than as an inducer of EMT [27]. Peinado et al. have proposed that Snail1 could be implicated in the initial migratory phenotype of primary tumours and considered it as an early marker of EMT, at least in ovarian tumours [10].

In the vast majority of the Snail1 positive ovarian carcinomas the nuclear protein was stained focally at rather low levels in the tumour epithelium and stroma. Nuclear content of Snail1 in epithelium or stroma in our material was not related to clinicopathological features, like tumour grade or stage of disease, nor had it prognostic significance. Relatively small material and short follow-up time of the patients could effect on this. However, nuclear staining was detected in borderline tumours suggesting that Snail1 might be an early factor in ovarian tumour development. Nevertheless, supporting our findings rather similar results have been reported [16]. In another ovarian cancer study using the material of 48 primary tumours nuclear Snail1 staining in epithelial cells in 38% of the primary tumours did not associate with any clinicopathological factors or overall survival [16].

Stromal cells are essential in tumourigenesis [28]. Presence of Snail1 positive stromal cells has previously been detected in various adeno and squamous cell carcinomas [14-19,21]. In our study, similar staining of Snail1 protein was observed both in stromal fibroblast-like fusiform cells and epithelial cells of ovarian carcinomas. Thus, a tumour may create its own stroma originating from malignant epithelium to facilitate tumour growth [29] and Snail1 could be a marker of these cells that have just undergone EMT [14]. Indeed, *in vitro *Snail1-transfected (squamous) carcinoma cells show complete EMT phenotype with fibroblastic characteristics [11]. However, in the present study expression of Snail1 in stromal cells was also detected in borderline tumours that do not have histological evidence of stromal invasion indicating that Snail1 may have also other functions in stromal fibroblast-like fusiform cells [27]. Snail1 has also been identified as a regulator of terminally differentiated mesenchymal cells [30], and further, during tumourigenesis, Snail1 expression in stromal cells may indicate interactions of fibroblasts with dedifferentiated tumour cells [19]. Snail1-induced EMT has been proposed also to accelerate cancer metastasis by enhancing invasion and by inducting immunosuppression [31]. Altogether, Snail1 expression in the stroma of borderline tumours may indicate importance of the microenvironment already in premalignant tumours and supports the role of tissue remodelling effect of Snail1.

In tumours, endothelial to mesenchymal transition (EndMT) is an important source of cancer-associated fibroblasts [32]. Some of the endothelial cell nuclei in this ovarian carcinoma study were positive for Snail1. Snail1 staining in a subset of endothelial cells has been reported in adeno and squamous cell carcinomas [15,18,19]. However, we found positive endothelial cells also in benign and borderline tumours as well as in normal ovaries. A previous report has demonstrated that angiogenic vessels can undergo EndMT [32]. Kokudo et al. showed that mouse endothelial cells can differentiate into mural cells, such as pericytes and/or smooth muscle cells, and that Snail1 is associated with the process [33]. Also in normal human ovaries new blood vessel formation and subsequent regression occurs in each hormonal cycle [34].

Besides nuclear protein content we have also analysed cytoplasmic staining of Snail1 protein in epithelial cells. Opposite to nuclear immunoreactivity, we observed decreased cytoplasmic expression of Snail1 in epithelium that was significantly associated with increasing tumour malignancy (p < 0.001). Transcription factor Snail1 activity is controlled by subcellular location and cytoplasmic form of the protein is not considered active [10]. Keeping this in mind, cytoplasmic staining may point to post-transcriptional regulation of the protein in epithelial ovarian carcinomas [10,21] and in pre-malignant tumours, as shown in the present material.

## Conclusion

Nuclear expression of Snail1 protein in epithelial ovarian tumours was increased during tumour progression from precursor lesions into carcinomas. Snail1 expression detected in borderline tumours suggests a role for Snail1 also in early ovarian tumourigenesis preceding tumour invasion and metastasis. In addition, stromal positivity of Snail1 in borderline and malignant tumours underlines the importance of tumour microenvironment during development and progression of ovarian tumours.

## Competing interests

The authors declare that they have no competing interests.

## Authors' contributions

HT, YS, V-MK and AM designed the study. MA and KH gathered the material. IV was responsible for methodology. LK did laboratory analysis. HT, YS, V-MK, RS and LK are responsible for acquisition of data. HT and MA did the statistical analysis. HT, YS, V-MK and AM interpreted data. HT, V-MK and AM wrote the manuscript. All authors read and approved the final manuscript.

## Pre-publication history

The pre-publication history for this paper can be accessed here:

http://www.biomedcentral.com/1471-2407/9/289/prepub

## References

[B1] Parkin DM Bray F Ferlay J Pisani P Global cancer statistics, 2002 CA Cancer J Clin 2005 55 74 108 10.3322/canjclin.55.2.74 15761078

[B2] Shih IM Kurman RJ Ovarian tumourigenesis: A proposed model based on morphological and molecular genetic analysis Am J Pathol 2007 164 1511 1518 10.1016/s0002-9440(10)63708-x PMC1615664 15111296

[B3] Bell DA Origins and molecular pathology of ovarian cancer Mod Pathol 2005 18 S19 S32 10.1038/modpathol.3800306 15761464

[B4] Landen CN Birrer MJ Sood AK Early events in the pathogenesis of epithelial ovarian cancer J Clin Oncol 2008 26 995 1005 10.1200/JCO.2006.07.9970 18195328

[B5] Auersperg N Wong AS Choi KC Kang SK Leung PC Ovarian surface epithelium: biology, endocrinology, and pathology Endocr Rev 2001 22 255 288 10.1210/er.22.2.255 11294827

[B6] Thiery JP Sleeman JP Complex networks orchestrate epithelial-mesenchymal transitions Nat Rev Mol Cell Biol 2006 7 131 142 10.1038/nrm1835 16493418

[B7] Tuhkanen H Anttila M Kosma VM Heinonen S Juhola M Helisalmi S Kataja V Mannermaa A Frequent gene dosage alterations in stromal cells of epithelial ovarian carcinomas Int J Cancer 2006 119 1345 1353 10.1002/ijc.21785 16642473

[B8] De Wever O Pauwels P De Craene B Sabbah M Emami S Redeuilh G Gespach C Bracke M Berx G Molecular and pathological signatures of epithelial-mesenchymal transitions at the cancer invasion front Histochem Cell Biol 2008 130 481 494 10.1007/s00418-008-0464-1 18648847 PMC2522326

[B9] Ansieau S Bastid J Doreau A Morel AP Bouchet BP Thomas C Fauvet F Puisieux I Doglioni C Piccinin S Induction of EMT by twist proteins as a collateral effect of tumor-promoting inactivation of premature senescence Cancer Cell 2008 14 79 89 10.1016/j.ccr.2008.06.005 18598946

[B10] Peinado H Olmeda D Cano A Snail, Zeb and bHLH factors in tumour progression: An alliance against the epithelial phenotype? Nat Rev Cancer 2007 7 415 428 10.1038/nrc2131 17508028

[B11] Takkunen M Grenman R Hukkanen M Korhonen M Garcia de Herreros A Virtanen I Snail-dependent and -independent epithelial-mesenchymal transition in oral squamous carcinoma cells J Histochem Cytochem 2006 54 1263 1275 10.1369/jhc.6A6958.2006 16899764

[B12] Vega S Morales AV Ocana OH Valdes F Fabregat I Nieto MA Snail blocks the cell cycle and confers resistance to cell death Genes Dev 2004 18 1131 1143 10.1101/gad.294104 15155580 PMC415638

[B13] Becker KF Rosivatz E Blechschmidt K Kremmer E Sarbia M Höfler H Analysis of the E-cadherin repressor snail in primary human cancers Cells Tissues Organs 2007 185 204 212 10.1159/000101321 17587826

[B14] Franci C Takkunen M Dave N Alameda F Gomez S Rodriguez R Escriva M Montserrat-Sentis B Baro T Garrido M Expression of Snail protein in tumor-stroma interface Oncogene 2006 25 5134 5144 16568079 10.1038/sj.onc.1209519

[B15] Rosivatz E Becker KF Kremmer E Schott C Blechschmidt K Höfler H Sarbia M Expression and nuclear localization of Snail, an E-cadherin repressor, in adenocarcinomas of the upper gastrointestinal tract Virchows Arch 2006 448 277 287 10.1007/s00428-005-0118-9 16328348

[B16] Blechschmidt K Sassen S Schmalfeldt B Schuster T Höfler H Becker KF The E-cadherin repressor Snail is associated with lower overall survival of ovarian cancer patients Br J Cancer 2008 98 489 495 10.1038/sj.bjc.6604115 18026186 PMC2361433

[B17] Blechschmidt K Kremmer E Hollweck R Mylonas I Höfler H Kremer M Becker KF The E-cadherin repressor Snail plays a role in tumor progression of endometrioid adenocarcinomas Diagn Mol Pathol 2007 16 222 228 10.1097/PDM.0b013e31806219ae 18043286

[B18] Zidar N Gale N Kojc N Volavsek M Cardesa A Alos L Höfler H Blechschmidt K Becker KF Cadherin-catenin complex and transcription factor Snail-1 in spindle cell carcinoma of the head and neck Virchows Arch 2008 453 267 274 10.1007/s00428-008-0649-y 18712413

[B19] Franz M Spiegel K Umbreit C Richter P Codina-Canet C Berndt A Altendorf-Hofmann A Koscielny S Hyckel P Kosmehl H Expression of Snail is associated with myofibroblast phenotype development in oral squamous cell carcinoma Histochem Cell Biol 2009 131 651 660 10.1007/s00418-009-0559-3 19198871

[B20] Peinado H Moreno-Bueno G Hardisson D Perez-Gomez E Santos V Mendiola M de Diego JI Nistal M Quintanilla M Portillo F Lysyl oxidase-like 2 as a new poor prognostic marker of squamous cell carcinomas Cancer Res 2008 68 4541 4550 10.1158/0008-5472.CAN-07-6345 18559498

[B21] Elloul S Silins I Trope CG Benshushan A Davidson B Reich R Expression of E-cadherin transcriptional regulators in ovarian carcinoma Virchows Arch 2006 449 520 528 10.1007/s00428-006-0274-6 17024425

[B22] Sobin LH Wittekind Ch editors International Union Against Cancer (UICC). TNM Classification of Malignant Tumours 2002 6 New York: Wiley-Liss 165 169

[B23] Tavassoli FA Devilee P editors World Health Organization Classification of Tumours. Tumours of the Breast and Female Genital Organs 2003 Lyon: IARC Press 113 45

[B24] Soini Y Salo T Satta J Angiogenesis is involved in the pathogenesis of nonrheumatic aortic valve stenosis Hum Pathol 2003 34 756 763 10.1016/S0046-8177(03)00245-4 14506635

[B25] Chen X Halberg RB Burch RP Dove WF Intestinal adenomagenesis involves core molecular signatures of the epithelial-mesenchymal transition J Mol Histol 2008 39 283 294 10.1007/s10735-008-9164-3 18327651 PMC2544376

[B26] Blanco MJ Barrallo-Gimeno A Acloque H Reyes AE Tada M Allende ML Mayor R Nieto MA Snail1a and Snail1b cooperate in the anterior migration of the axial mesendoderm in the zebrafish embryo Development 2007 134 4073 4081 10.1242/dev.006858 17965052

[B27] Barrallo-Gimeno A Nieto MA The snail genes as inducers of cell movement and survival: Implications in development and cancer Development 2005 132 3151 3161 10.1242/dev.01907 15983400

[B28] Liotta LA Kohn EC The microenvironment of the tumour-host interface Nature 2001 411 375 379 10.1038/35077241 11357145

[B29] Petersen OW Nielsen HL Gudjonsson T Villadsen R Rank F Niebuhr E Bisell MJ Ronnov-Jessen L Epithelial to mesenchymal transition in human breast cancer can provide a nonmalignant stroma Am J Pathol 2003 162 391 402 12547698 10.1016/S0002-9440(10)63834-5 PMC1851146

[B30] Rowe RG Li XY Hu Y Saunders TL Virtanen I Garcia de Herreros A Becker KF Ingvarsen S Engelholm LH Bommer GT Mesenchymal cells reactivate Snail1 expression to drive three-dimensional invasion programs J Cell Biol 2009 184 399 408 10.1083/jcb.200810113 19188491 PMC2646556

[B31] Kudo-Saito C Shirako H Takeuchi T Kawakami Y Cancer metastasis is accelerated through immunosuppression during Snail-induced EMT of cancer cells Cancer Cell 2009 15 195 206 10.1016/j.ccr.2009.01.023 19249678

[B32] Zeisberg EM Potenta S Xie L Zeisberg M Kalluri R Discovery of endothelial to mesenchymal transition as a source for carcinoma-associated fibroblasts Cancer Res 2007 67 10123 10128 10.1158/0008-5472.CAN-07-3127 17974953

[B33] Kokudo T Suzuki Y Yoshimatsu Y Yamazaki T Watanabe T Miyazono K Snail is required for TGFβ-induced endothelial-mesenchymal transition of embryonic stem cell-derived endothelial cells J Cell Sci 2008 121 3317 3324 10.1242/jcs.028282 18796538

[B34] Ramakrishnan S Subramanian IV Yokoyama Y Geller M Angiogenesis in normal and neoplastic ovaries Angiogenesis 2005 8 169 182 10.1007/s10456-005-9001-1 16211363

